# In Vitro Dissolution and in Silico Modeling Shortcuts in Bioequivalence Testing

**DOI:** 10.3390/pharmaceutics12010045

**Published:** 2020-01-04

**Authors:** Moawia M. Al-Tabakha, Muaed J. Alomar

**Affiliations:** 1 Department of Pharmaceutical Sciences, College of Pharmacy and Health Sciences, Ajman University, P.O. Box 346, Ajman, UAE; 2 Department of Clinical Sciences, College of Pharmacy and Health Sciences, Ajman University, P.O. Box 346, Ajman, UAE; muayyad74@yahoo.com

**Keywords:** bioequivalence, in silico pharmacokinetic simulations, similarity factor, dissolution, publication bias, biowaiver, biopharmaceutics classification system

## Abstract

Purpose: To review in vitro testing and simulation platforms that are in current use to predict in vivo performances of generic products as well as other situations to provide evidence for biowaiver and support drug formulations development. Methods: Pubmed and Google Scholar databases were used to review published literature over the past 10 years. The terms used were “simulation AND bioequivalence” and “modeling AND bioequivalence” in the title field of databases, followed by screening, and then reviewing. Results: A total of 22 research papers were reviewed. Computer simulation using software such as GastroPlus™, PK-Sim^®^ and SimCyp^®^ find applications in drug modeling. Considering the wide use of optimization for in silico predictions to fit observed data, a careful review of publications is required to validate the reliability of these platforms. For immediate release (IR) drug products belonging to the Biopharmaceutics Classification System (BCS) classes I and III, difference factor (*ƒ*_1_) and similarity factor (*ƒ*_2_) are calculated from the in vitro dissolution data of drug formulations to support biowaiver; however, this method can be more discriminatory and may not be useful for all dissolution profiles. Conclusions: Computer simulation platforms need to improve their mechanistic physiologically based pharmacokinetic (PBPK) modeling, and if prospectively validated within a small percentage of error from the observed clinical data, they can be valuable tools in bioequivalence (BE) testing and formulation development.

## 1. Introduction

To classify drugs based on their aqueous solubility and intestinal permeability, the Biopharmaceutics Classification System (BCS) represents a cornerstone for four-category classification. The BCS categories are: class I (high solubility, high permeability), class II (low solubility, high permeability), class III (high solubility, low permeability), and class IV (low solubility, low permeability) [1]. BCS is of particular significance because it is required in any new drug application (NDA) and allows for biowaivers of classes I and III drugs when applying for abbreviated new drug application (ANDA) [2]. Therefore, this system has been widely used since its inception in 1995 by Amidon et al., with adoption by various regulatory agencies [3]. Normally, for establishing bioequivalence, the calculated 90% confidence interval of the ratios for area under the drug concentration-time curve (AUC) and maximum concentration (C_max_) of the test and reference products should fall within 80% to 125% of the point estimate [4,5].

Prior to 2015, the US Food and Drug Administration (FDA) only considered biowaivers for class I drugs. Thereafter, the FDA biowaiver to class III drugs was a convergence with other health legislative authorities, including the European Medicines Agency (EMA) and the World Health Organization (WHO) [6]. Thereby, by applying the most conservative guidelines for bioequivalence (BE) testing, it would be feasible to obtain marketing authorization in different jurisdictions, including emerging regulators.

Biowaivers avoid unnecessary use of human testing, facilitate access to different markets, save time, and effectively reduce development costs. The biowaiver is based on the idea that two drug formulations/products are bioequivalent when: (i) they are immediate-release (IR) and act as oral solutions within the gastrointestinal (GI) tract, (ii) there is no precipitation of the active pharmaceutical ingredient (API) once dissolved in the GI tract, and (iii) the formulations exhibit rapid or very rapid in vitro dissolution using the recommended test methods [6]. The FDA further requires that for class I drug formulations, excipients will not affect the rate or extent of drug absorption, while for class III drug formulations, the test product is qualitatively the same and quantitatively very similar to the reference [7].

Other than BE testing of generic drug products compared to a reference, a newly marketed product needs to be bioequivalent with the clinical-scale formulation undertaken in phase III trials. This also applies to post-approval changes in the commercialized product. Different scenarios exist for BE testing, such that potential for biowaivers exists. In class III drugs, if the excipients used in the formulations do not influence absorption and the dissolution is very rapid, then there is no reason for them not to be bioequivalent [6]. To provide evidence, the difference factor (*ƒ*_1_) and similarity factor (*ƒ*_2_) can be used. If the drug dissolution from the tested products is at least 85% within 15 min in different dissolution media, then these factors need not be calculated, as the products’ bioequivalence is self-evident, because the drug formulation will be in solution form once it reaches the duodenum [4]. There has been a debate as to whether the point estimate *ƒ*_2_ is reliable when there is a significant batch-to-batch variation, and bootstrap confidence intervals for *ƒ*_2_ have therefore been suggested [8].

**Figure 1:**
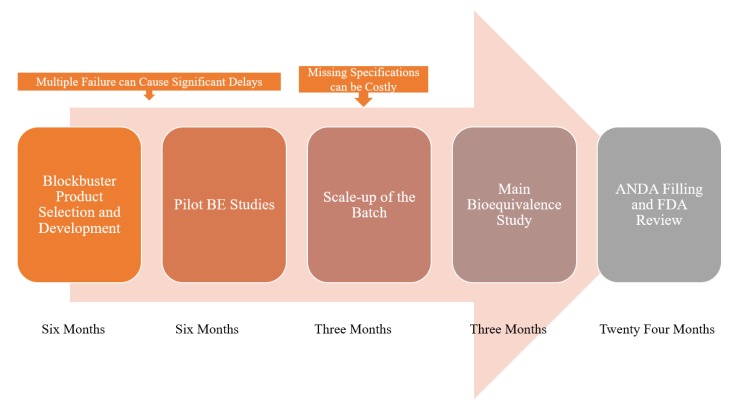
Scheme showing the development process of a generic product and where the delays can be circumvented using in silico platforms.

In silico simulation programs have taken a significant role in NDAs and ANDAs in recent years based on physiologically-based pharmacokinetic models (PBPK) that provide a mechanistic framework for prediction of drug exposure in humans [9,10]. In silico simulations have been suggested to reduce time and effort in developing generic drug products (see Figure 1).

In silico platforms include SimCyp^®^ (a Certara company, Sheffield, UK), which uses PBPK modeling and simulation capabilities for virtual BE. Specialized software such as nonlinear mixed-effects modeling (NONMEM^®^, ICON Plc., Dublin, Ireland), and the mechanistic oral absorption modeling and simulation PK-Sim^®^ (Bayer Technology Services GmbH, Leverkusen, Germany) allow extrapolation of plasma concentration-time profiles from the in vitro data. These platforms will not only help to reduce time, but also support biowaiver application.

The aforementioned models have not been studied thoroughly against their claims. One study indicated that differences between observed and simulated C_max_ and AUC which are typically used in BE study can be variable, and as large as 10 folds, which is much higher than the typically accepted difference [11]. Variability within twofold is commonly accepted; however, recommendations were to limit this to 1.25 folds [12].

Parameter fitting in PBPK modeling is an essential part of model refinement, provided that it can be justified. While having details of codes and data are essential for the reproducibility of published data, the complexity of databases and algorithms within commercial platforms do not allow for publishing all the details. However, if details of parameters in compound and population models are published, the study can be reproduced using the specific platform. On the other hand, there have been many publications using easy parameter fitting not substantiated by reliable evidence and falsely showing the fitted results as “prediction”. In addition, the model equations and physiological parameters have often not been completely disclosed, suggesting that many articles have been published without appropriate peer review.

Since these platforms are different and are continuously updated to tune and improve their prediction power [13], a careful review of these models is warranted to provide information regarding the extent to which they can be useful. Therefore, the aims of this work were to systematically review the in silico platforms for their reliability in providing bioavailability (BA) information about drug products and to evaluate the suitability of the point estimate *ƒ*_2_ in support of biowaiver applications.

## 2. Search Methodology

The preparation of this systematic review followed the Preferred Reporting Items for Systematic Reviews and Meta-Analyses (PRISMA) guidelines [14]. The databases used for the literature review were PubMed and Google Scholar. The timeline for the reviewed studies covered the past 10 y period. The terms used in searching PubMed and Google Scholar were required to be in the title of the manuscript. These terms were: “simulation AND bioequivalence” and “modeling AND bioequivalence” in the English language. Publications from the literature review were additionally screened in all databases used depending on the abstracts to exclude results not directly addressing BE in vitro, or not using simulation/modeling programs for bioequivalence testing such as statistical methods only. For the purpose of critically reviewing the resulting literature, the search was later extended to other publications.

## 3. Literature Reviewed

**Figure 2:**
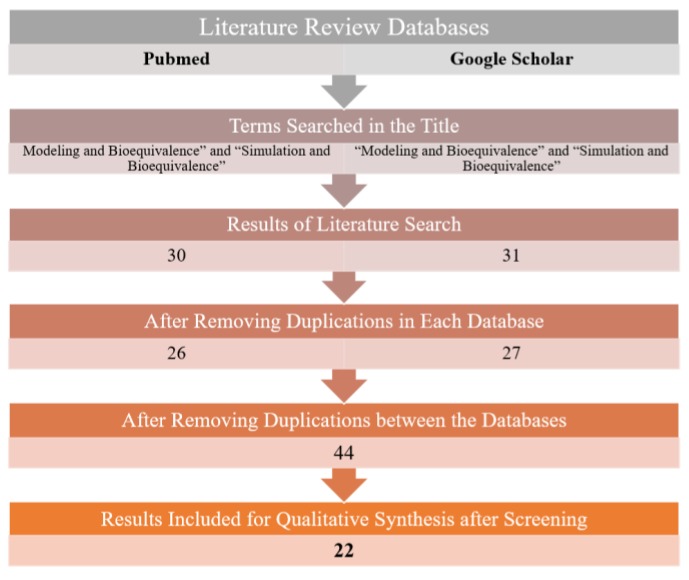
Results from literature review for the terms “modeling and bioequivalence” and “simulation and bioequivalence” using Pubmed and Google Scholar databases in accordance with PRISMA guidelines.

Searching the databases yielded a total of 22 results after removing duplications and screening the articles in accordance with the PRISMA flowchart. Figure 2 shows the outcomes of the literature search.

The review of the 22 articles showed that among simulation platforms, GastroPlus™ (Simulation Plus, Lancaster, CA, USA) was used most frequently followed by SimCyp^®^. Similarity and difference factors from the in vitro dissolution studies were also common (see Table 1 for more details).

## 4. Similarity and Difference Factors

The model-independent mathematical approach was introduced by Moore and Flanner in 1996 included the calculation of difference factor (*ƒ*_1_) and similarity factor *(ƒ*_2_) to compare the dissolution profiles of the different generics, and generics to branded drug products [36]. The relative difference between two curves *ƒ*_1_ values up to 15% indicates little difference between two dissolution curves, while drug products are considered similar when the calculated *ƒ*_2_ is between 50 to 100 [4]. In one study, *ƒ*_1_ and *ƒ*_2_ and PBPK simulations for atorvastatin formulations proved to be bioequivalent, supporting the argument for class II inclusion in biowaiver applications, ideally in silico modeling in conjunction with in vitro dissolution [37]. However, for class II drug products, the probability of BE after a similar dissolution profile was 61% with false positive results (similar dissolution but not bioequivalent (NBE)) was up to 90% [38,39].

In vitro dissolution studies are essential in BE testing; however, studies indicated that the lower limit in the *ƒ*_2_ (i.e., 50) should be further lowered to match observed in vivo BE results [4,16,22,24]. Moreover, the one-point estimate for *ƒ*_2_, together with the dissolution model-independent approach (i.e., not being mechanistic in nature) are considered drawbacks of this approach. Islam and Begum suggested the use of the conservative bootstrap confidence intervals for dissolution similarity because of the limitations of the point estimate *ƒ*_2_ [8]. They used non-parametric and parametric methods for the construction of confidence intervals and found them both to yield similar results. The point estimate *ƒ*_2_ can still be valid if the data variability is low, otherwise much more than 12 units would be necessary to run the dissolution experiment. Alternative model-dependent and independent methods can also be used to assess dissolution similarity when data variability is high, as when within-batch coefficient of variation (CV) is >15% [40]. These include the most commonly cited by regulatory agencies, the multivariate statistical distance (MSD). It can be determined using the raw dissolution data with or without time variables as parameters [41].

## 5. GastroPlus™

GastroPlus™ is available with different modules depending on the area of investigation, making it a versatile application software. Simulations using GastroPlus™ were claimed to support biowaiver for class III drugs such as cimetidine, atenolol, and amoxicillin, which could also be extended to other class III drugs [27]. For lesinurad, a class II drug, it was claimed that the FDA accepted the control strategy for the IR tablets based on product dissolution and particle size specifications using GastroPlus™ PBPK modeling without the need for BA study [9,28]. However, careful examination of the Pepin et al. study showed that the adjusted theoretical particle size distribution of lesinurad based on in vitro dissolution profiles was unlikely to be realistic [28]. In this respect, they nevertheless used the theoretical particle size as input into GastroPlus™. They justified the unusual theoretical particle size distribution as likely to be the impact of the granulation and disintegration process. Recent publications suggest that the FDA is more prudent regarding the use of modeling and simulation for a regulatory purpose, because of the need to establish the model’s “credibility” for its intended use [42].

**Figure 3:**
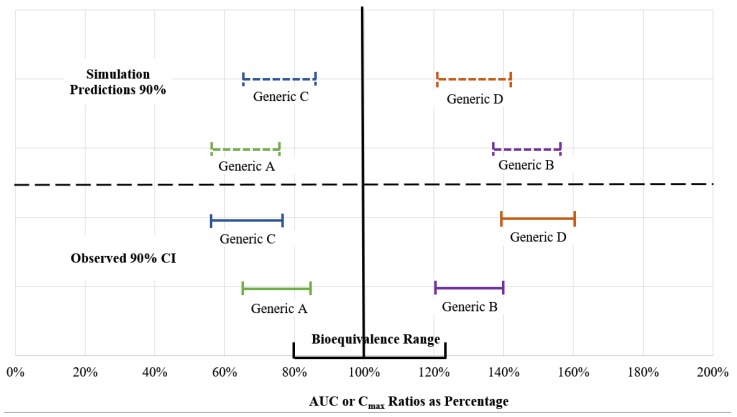
The false predictions of bioequivalence based on the simulation that accept up to 1.25-fold differences between predicted and observed AUC or C_max_ ratios.

In agreement with the review of Li et al., GastroPlus™ was the most widely used platform [43]. Their recent review of the predictive performance of PBPK models using different platforms (i.e., GastroPlus™, SimCyp^®^, and STELLA) for the effect of food on oral drug absorption showed that among the 48 food effect predictions, 50% were predicted within 1.25 fold of observed values, and 75% within 2 fold [43]. Even the more rigorous 1.25-fold variability with the observed values could falsely lead to BE or NBE conclusions (Figure 3). The rationale to use of 1.25 fold was to be in concordance with the accepted bioequivalence 80 to 125% range from the reference product. Furthermore, by careful examination of the graphic presentation of datasets for predicted versus observed values of AUC, C_max_ and time to maximum concentration (t_max_) ratios, in the Li et al. review, it was evident that there was little or no reliable prospective predictability. The authors concluded that the experience to draw any conclusion is still inadequate.

GastroPlus™ was employed to predict food effects on generic and reference class II drug formulations [23]. The predicted food effects were up to 10% prediction error and the virtual BE study confirmed that the products were bioequivalent. Although the authors used 1000 mL of media for their dissolution studies, they stated that sink conditions were not achieved. Nevertheless, they used the dissolution data for input into the GastroPlus™. Furthermore, because of the limited data available, they assumed the bioavailability to be 40%, and consequently had to lower clearance and volume of distribution in the PKPlus™ module.

Mitra et al. investigated the reliability of GastroPlus™ and the PKPlus™ module to simulate plasma concentration vs. time profiles for drugs of classes I and II [44]. The prediction for both classes closely matched the observed data, claiming that PK parameters were predicted accurately. Hence, the model was considered acceptable for predicting successes of BE based on dissolution data. However, they modified or used default values for absorption scale factor (ASF), effective permeability (P_eff_) and/or fluid in the gastrointestinal (GI) tract to fit observed data which makes the reliability of GastroPlus™ questionable. In this respect, Mitra et al. modified the ASF in the duodenum of human fasted physiological model from the default of 2.794 to 3.794 (136% increase) to better fit the observed data from absolute bioavailability using 120-mg oral dose and 25-mg IV dose [18]. They justified the change as within possible ranges for in vivo deviation of parameters merely relying on in vitro measurements.

The use of unreliable data is common in publications involving simulations. For example, to simulate plasma concentrations, input data was used from the different knowledgebase, including predictions from ADMET Predictor™, a module in the GastroPlus™, such as the P_eff_ [24]. In this respect, fitting of the data was common [28,35,45,46] and could be unrealistic [46]. Model equations and physiological parameters were not given and only stated as “default” [46,47]. These inconsistencies in using GastroPlus™ for predicting BE profiles, coupled with the easy fitting of parameters as with other platforms may cause the results to be subjective in nature and unreliable. On the other hand, the use of default parameters within a commercially available platform is usually part of the advantage of using such platforms, with databases and parameters that have been extensively researched and validated. To validate GastroPlus™ properly, a prospective study is needed whereby results from the platform are subsequently verified by in vivo observations to be within acceptable difference, which is much less than commonly cited in the literature. This requirement is not fulfilled by the study of Basu et al., where the dissolution profile predictions from Dose Disintegration and Dissolution Plus (DDDPlus™), a piece of software that can be integrated with the GastroPlus™ platform, were retrospectively compared to the reported ANDA for metoprolol test and reference ER formulations [26].

One claimed benefit of GastroPlus™ is enabling mechanistic comprehension of IVIVC under different conditions [48]. For example, a rifampicin-loaded solidified self-nanoemulsifying drug delivery system demonstrated a good IVIVC level A with a predicted systemic absorption of 96.5% using IVIVCPlus™ module of GastroPlus™ [46]. For this, physicochemical input parameters were experimental, literature, or ADMET Predictor^®^-based and deconvolution and then convolution to generate predictions and reconstruct the plasma drug concentration curve was applied. IVIVCPlus^®^ module in GastroPlus™ was also used for IVIVC modeling using deconvolution methods to obtain in vivo dissolution profiles. Although this method is traditionally used, it is subject to both mechanistic and statistical concerns. Deconvolution methods work better when the drugs completely follow first-order PK [49].

The PBPK simulation model GastroPlus™ was used to support FDA application to authorize lesinurad (class II drug) marketing as well as few other drugs without the need for bioavailability (BA) study [9]. However, the published literature and perhaps also the results from submissions to the FDA may be biased, because only the “good” results tend to be published or submitted, whereas the “true” picture may be lost. It is always important to consider the “publication bias” when interpreting the results, especially when the knowledgebase is still small.

## 6. SimCyp^®^

**Figure 4:**
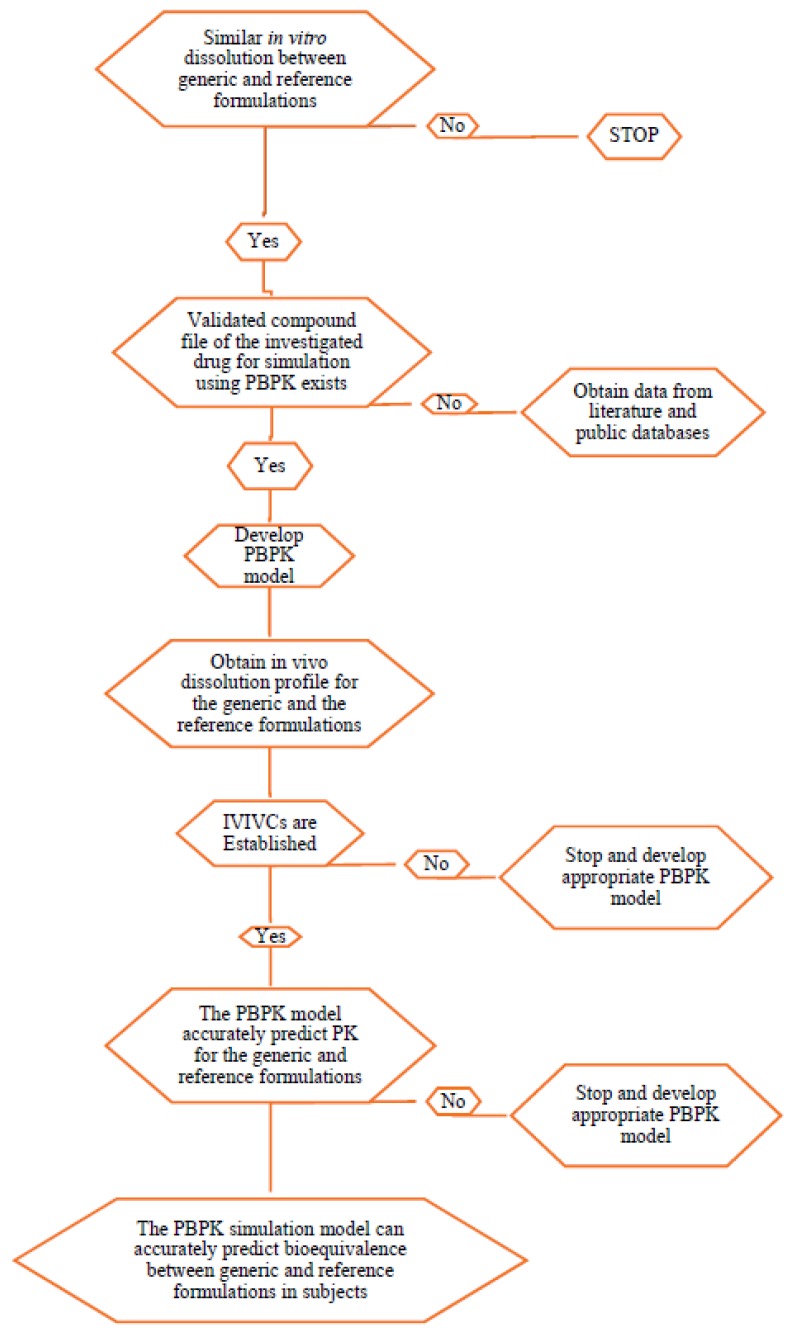
Typical workflow for virtual bioequivalence studies using the PBPK models.

The population-based simulator of SimCyp^®^ includes demographic, physiologic and genomic databases to account for patient variability. Typical simulation uses of SimCyp^®^ are provided in the flowchart of Figure 4. The effect of drug-drug interactions (DDIs), mediated by cytochrome P450 isoenzymes modulators, were assessed using Simcyp^®^ and was claimed to be capable of testing DDIs in untested scenarios [50]. However, default models were used in the software and applied selective distribution PBPK models depending on the drug. They accepted clearances and AUCs within two folds of the observed values. This can potentially lead to wrong conclusions in comparison to the more rigorous limit of 1.25 fold. Wagner et al. studied the effects of CYP3A inducers on substrate exposures based on FDA PBPK submissions between 2008 and 2014 [12]. They found that the only PBPK software used was the SimCyp^®^ in all submissions and that the software’s default rifampicin model as strong inducer would need to be modified in order to improve AUC and C_max_ ratios for predicted over-observed values.

Since some drugs’ bioavailability might be affected by coadministration with drugs that elevate stomach pH, Fan et al. studied the effect of proton pump inhibitor (PPI) on prasugrel HCl product bioequivalence using Simcyp^®^ [32]. The platform was not able to distinguish between the drug salt and the base present in different proportions in the tested formulations and therefore not able to predict the whole C_max_ based on one deconvoluted intrinsic base solubility. Hence, a series of relationships between C_max_ and the base content was predicted based on the deconvoluted base solubilities of formulations containing different proportions of the base. Failure in BE testing was more probable when the base content in the formulations exceeded 20% (i.e., the salt is <80%). The authors used predicted values from ADMET predictor^®^ in GastroPlus™ and fitted values based on the observed plasma concentration data. They assumed the gastric pH of the fasting stomach to be 1.2, the absorbed fraction is the same regardless of base proportion, and that no interaction occurs between the base and the salt. These can be black boxes in assessing the reliability of the platform.

The concentration-time profile of perampanel was also claimed to be adequately predicted in another study focusing on the CYP3A inhibitor ketoconazole effect as a case for DDI [51]. The authors, however, used SimCyp^®^ to gain additional information in phase I clinical study. Additionally, they included the intrinsic clearance retrospectively from the observed clinical data to allow better AUC prediction.

The bioequivalence of levothyroxine and nifedipine formulations was studied utilizing Simcyp^®^ based on PBPK modeling framework in achlorhydria conditions such as in the elderly [31]. In vitro dissolution profile in neutral pH was used as input into the model. Unlike levothyroxine (class III drug), good virtual IVIVC was obtained for nifedipine (class II drug), irrespective of pH. The IVIVCs were between in vitro dissolution at different pH and the deconvoluted in vivo dissolution obtained by the platform from plasma concentration profiles in fasted and fed state. It has been shown that deconvolution is an unstable process and associated with several statistical limitations [52]. The authors nevertheless concluded it is important to establish IVIVC and that after PBPK model verification from bioequivalence clinical data in healthy volunteers, it will be possible to use virtual bioequivalence study in specific patient populations. In their study, several drugs’ physicochemical values were either default or predicted in Simcyp^®^, which may reduce the reliability of the results.

Correlation coefficients between SimCyp^®^-predicted concentration profiles and the observed plasma concentrations of rusovastatin (BCS class III drug) after oral administration were described as good ranging between 0.85 and 0.87 [53]. However, the study involved searching for the best parameter values for P_eff_ estimation that produce plasma concentration matching the observed values. It was also reported that SimCyp^®^ is capable of predicting PK parameters of the elderly population for a variety of drugs [54]. While the defaults of the platform, such as liver volume and blood flows and enzyme status, are for the North European Caucasian population, it allows changes to parameters based on other populations. The authors provided equations for the calculation of PK parameters and the description of changes in the elderly population. However, to validate the SimCyp^®^ predictions with the observed data for orally administered seven drugs, they claimed that clearances were predicted satisfactorily even though some drugs’ predicted clearances were within 1.5 folds of the observed values.

In silico platforms Simcyp^®^, GastroPlus™, and GI-Sim (AstraZeneca internal tool) were examined for performance by comparing the predicted plasma concentration-time profiles [55]. The prediction accuracy was based on the absolute average fold error (AAFE) for the PK parameters C_max_, time to maximum concentration (t_max_), and AUC. For AUC, the AAFE values ranged from 1.7–2.2, 1.5–1.6, and 1.3–1.4 for Simcyp^®^, GastroPlus™, and GI-Sim, respectively with a tendency for underprediction of AUC and Cmax using Simcyp^®^ and GastroPlus™. Despite the authors claiming that GastroPlus™ and GI-Sim performed better than Simcyp^®^ several pitfalls were noted. These include the use of default drug parameters, issues with estimating pharmacokinetic (PK) parameters from fraction absorbed, and the operator bias in selecting input parameter values and in model options in the platforms [56].

## 7. NONMEM^®^

The nonlinear mixed-effects modeling (NONMEM^®^) was developed starting from the 1970s by Lewis Sheiner and Stuart Beal at the University of California. Initially, it was intended for the analysis of PK, but now has been extended to be a tool for population PK/pharmacodynamic (PD) analysis [57]. The virtual simulations of NONMEM^®^ to study bioequivalence using single and multiple-dose administration to predict C_max_ and steady-state concentrations (SSC), respectively showed that while single-dose administration resulted in significant differences, they might not be sensitive to predict failure in BE for steady-state concentrations [58]. These results were also supported by Kim et al. [35]. Kim et al. needed to use absorption lag time and fix the maximal nonlinear clearance in NONMEM^®^ for the PK model to better fit the observed values.

Cuesta-Gragera et al. tested the validity of NONMEM^®^ for an adapted semi-physiological model of acetylsalicylic acid [19]. They used pharmacokinetic parameters input from literature to validate concentration-time curves obtained and found them to be closely predicting the actual published experimental data. The results of C_max_ ratios were more sensitive to the change in the in vivo dissolution rate constant than did the AUC ratios. Metformin (class III drug) was also examined for model fitting in NONMEM^®^ and was found to be best described by two-compartment as it provides an adequate agreement between predicted and observed concentration values [34]. Their study simulation study results were compared retrospectively to clinical data available internally in their company Novartis to show that country drug source or race has a negligible effect on the PK parameters. This could benefit the company’s argument that there is no need for additional bioequivalence studies with multiple countries’ reference products if bioequivalence is established with one country. Metformin formulations were also assessed for their bioequivalence by developing a population PK model using NONMEM^®^ based on comparative PK study of a single agent and a fixed-dose combination of metformin in 36 healthy Korean volunteers. It was found to be best described by a two-compartment model, first-order elimination with two absorption processes separated by a lag time [59]. The study, however, concluded that actual clinical trials should be conducted and that simulations would be useful only to identify possible differences in formulations.

NONMEM^®^ was used to simulate concentration-time profiles of liposome-encapsulated doxorubicin and free doxorubicin based on observed concentrations after in vivo administration of the innovator product [60]. They were well characterized by a one- and two-compartment model, respectively. The purpose of the simulation study was to find the most discriminatory metric to the difference between doxorubicin release from the test and reference product. The platform was also used to generate Monte Carlo simulations of all bioequivalence studies to determine which analyte (whether parent drug or any of its active metabolites) is most sensitive to formulation changes and results from different scenarios demonstrated the analyte to be the parent drug [61]. Formulation changes were made through altering in vivo dissolution, which indicates that the platform can be subject to optimization by the operator. In another study, the simulated concentration-time profiles following formulation changes obtained by GastroPlus™ were used as pharmacokinetic input into NONMEM^®^ to determine if pharmacokinetics changes would affect the metoprolol pharmacodynamics significantly [62]. However, it was found that no significant therapeutic effect differences were produced despite pharmacokinetic changes, but this was attributed to the insensitivity of the endpoint used. The study nevertheless demonstrates that predictions from one platform were used in another platform.

Because traditional IVIVCs based on deconvolution/convolution assume linearity in PK, they cannot be applied to drugs exhibiting non-linear PK. In this respect, Gaynor et al. suggested the use of a compartmental approach based on differential equations using NONMEM^®^ to conveniently fit the nonlinearity with speed computer run-time [63]. They used a convolution approach which is superior to deconvolution; however, they simulated the data so that strong IVIVC would exist to serve their study purpose.

## 8. PK-Sim^®^

PK-Sim^®^ is a tool for body PBPK modeling in humans and the common laboratory animals such as mouse, rat, minipig, dog, and monkey. The model considers dosage form dependent GI transit, disintegration, and dissolution processes of various IR and ER dosage forms [13].

In a study, PK-Sim^®^ was used to assess the performances of seven generic products of 25 mg carvedilol (class II drug) tablets in the Uruguayan market [64]. The in vitro drug release was shown to affect carvedilol predicted C_max_, demonstrating the need for similar in vivo bioequivalence testing of other commonly used drugs. The authors, however, used the unorthodox dissolution USP-4 apparatus results as input into the platform, justifying this as a more biorelevant scenario for in vitro comparison. They also used physicochemical, PK, pharmacodynamic parameters reported in the literature and the platform defaults for anatomical and physiological parameters variability between subjects and for transit times across the GIT.

To estimate azithromycin (class I drug) P_eff_ in PK-Sim^®^, the best values that would produce concentration-time profiles matching the observed was used [65]. While doing this, the authors claimed that there was a good fit between the observed and predicted concentration profiles. Although BE studies usually recruit a small number of healthy subjects, they are calculated to detect any difference in the BE metrics. The authors in their clinical study used only eight subjects without indicating its study power, while regulatory agencies require a minimum of 12 subjects [66]. PK-Sim^®^ was claimed to be useful in separating the confounding factors of intestinal and first-pass metabolism that would affect the deconvoluted in vivo dissolution data traditionally used in IVIVC; however, little information was obtained regarding its use [67].

## 9. Other in Silico Platforms

Other simulation models exist, such as generic PBPK mode using MATLAB^®^ software (MathWorks, Inc., München (Ismaning), Germany.). It was used for additional investigation of the output from NONMEM^®^, model simulations, and statistical and graphical analyses [68]. The GI-Sim model was used to investigate the absorption of 12 APIs chosen because of permeability/dissolution limitations [69]. The authors claimed that good predictions were obtained by GI-Sim for >95% of the drugs, as values of C_max_ and AUC were within 2 folds of the observed clinical data. Not only could two folds be considered insipid for describing the GI-Sim as having “good” predictions, but two of the authors were also from the same GI-Sim-owned company.

Several other platforms have been discussed by other researchers [13,70,71,72]; however, they did not gain as much popularity as the platforms presented in the review. Regulatory agencies have shown interest in better understanding the simulation platforms’ reliability in dossier applications with consistency in reporting [73]. In 2012, the European Union (EU) initiated work packages under the project oral biopharmaceutics tools (OrBiTo) to improve oral absorption prediction tools based on formulation variables [74]. The FDA internal research initiatives included a potential grant for the development of a virtual bioequivalence trial simulation platform that integrates population pharmacokinetic modeling algorithms into PBPK models [75].

## 10. Conclusions

The BCS is well established as an approach for biowaivers, therefore not requiring in vivo BA and BE studies for class I and III IR solid oral dosage forms. GastroPlus™ appears to be the most widely used platform that may benefit drug development companies by reducing their time, efforts and associated costs. Nevertheless, publication biases and fitting methods prevent distinguishing the merits of one platform over others present in this review. Continuous development of in silico methods will ensure enhanced predictability in comparison to actual BE testing. This can be achieved by a more complexed mechanistic approach that is capable of accounting for a variety of confounding factors that are associated with the PBPK modeling and eliminating modeler bias in the selection of input parameter values. Validated platforms with prospective predictivity less than the recommended 1.25 fold of the observed values are needed. Publications tend to optimize the fitting of different parameters to match observed ones retrospectively and assume predictions within two folds to be satisfactory. These make the current in silico platforms black boxes. Regulatory agencies need to be assured of the validity of the simulation platforms and experiences in this field are still immature. The strict *ƒ*_2_ values used in biowaiver applications should be revised to reflect in vivo performances of drug products, while other mechanistic and model-independent approaches should be explored, especially when the in vitro dissolution data tend to vary considerably.

## Figures and Tables

**Table 1:** Frequency of in vitro and in silico use of results to support of bioequivalency tests.

In Vitro/In Silico	Number of Times Used in the Reviewed Literature
Similarity/Difference factors (*ƒ*_1_/*ƒ*_2_)	5 [15,16,17,18,19]
GastroPlus™	12 [13,18,20,21,22,23,24,25,26,27,28,29]
SimCyp^®^	6 [13,15,23,30,31,32]
NONMEM^®^	4 [19,33,34,35]
PK-Sim^®^	2 [13,30]

The following in vitro and in silico tools for bioequivalence testing were reviewed.

## Abbreviations

AAFE	Absolute average fold error
ANDA	Abbreviated new drug application
API	Active pharmaceutical ingredient
ASF	Absorption scale factor
AUC	Area under drug concentration-time curve
BA	Bioavailability
BE	Bioequivalence
BCS	Biopharmaceutics Classification System
C_max_	Maximum plasma drug concentration
CV	Coefficient of variation
DDIs	Drug-drug interactions
EMA	European Medicines Agency
ER	Extended-release
EU	European Union
*ƒ*_1_	Difference factor
*ƒ*_2_	Similarity factor
FDA	Food and Drug Administration
GI	Gastrointestinal
GIT	Gastrointestinal tract
IR	Immediate release
IV	Intravenous
IVIVC	In vitro-in vivo correlation
MR	Modified release
MSD	Multivariate statistical distance
NBE	Nonbioequivalence
NDA	New drug application
NONMEM	Nonlinear mixed effects modeling
OrBiTo	Oral biopharmaceutics tool
PBPK	Physiologically based pharmacokinetic
PD	Pharmacodynamic
P_eff_	Effective permeability
PK	Pharmacokinetic
PPI	Proton pump inhibitor
PRISMA	Preferred Reporting Items for Systematic Reviews and Meta-Analyses
SSD	Steady-state concentrations
USP	United States Pharmacopeia
WHO	World Health Organization
